# Antioxidant Potential of Plumieride against CCl_4_-Induced Peroxidative Damage in Rats

**DOI:** 10.3390/antiox3040798

**Published:** 2014-11-27

**Authors:** Dharmendra Singh, Priya Vrat Arya, Ashutosh Sharma, Ved Prakash Aggarwal, Mahabeer Prasad Dobhal, Radhey Shyam Gupta

**Affiliations:** 1 Centre for Advanced Studies, Department of Zoology, University of Rajasthan, Jaipur 302 055, India; E-Mail: d.singh2009@gmail.com; 2 Department of Zoology, Dyal Singh College, University of Delhi, Lodhi Road, New Delhi 110 003, India; E-Mails: zoology.dsc@gmail.com (P.V.A.); vpa541@gmail.com (V.P.A.); 3 Department of Chemistry, University of Rajasthan, Jaipur 302 055, India; E-Mails: ashutoshs_912@rediffmail.com (A.S.); mpdobhal@yahoo.com (M.P.D.)

**Keywords:** antioxidants, carbon tetrachloride, free radicals, marker enzymes, plumieride

## Abstract

In search of a new potent as an antioxidant from natural sources, plumieride—an iridoid isolated from the methanol extract of the bark of *Plumeria bicolor* (family Apocynaceae) was evaluated for its antioxidant potential against CCl_4_-induced peroxidative damage in liver of rats. The antioxidant potential was evaluated by using hepatic tissue for SOD (superoxide dismutase), CAT (catalase), GSH (reduced glutathione), GPx (glutathione peroxidase), GR (glutathione reductase) and LPO (lipid peroxidation) alongwith the concomitant blood serum for AST & ALT (aspartate and alanine transaminases), GGT (gamma glutamyl transpeptidase), ALP (alkaline phosphatase), total bilirubin and total protein contents. All the biochemical parameters were significantly (*p* ≤ 0.001) altered by CCl_4_ (0.3 mL/kg body weight/twice a week, intra-peritoneally for 30 days). Simultaneously, oral treatment with plumieride (5, 10 and 20 mg/kg body weight/day for 30 days), restored all the parameters towards a normal level, remarkably. The histological findings of liver sections further corroborated the antioxidant potential of plumieride compared with standard drug-silymarin. In conclusion, plumieride consists of sugar molecules, which have alcoholic groups. Therefore, the alcoholic groups of sugar increase its antioxidant potential through intermolecular hydrogen bonding along with the thiol(SH) group of non-protein thiols and enzymes resulting in the restoration of the antioxidant system. Therefore, it might be considered a natural antioxidant against peroxidative damage in rats.

## 1. Introduction

Free radicals contribute towards tissue injury through covalent binding and lipid peroxidation whereas the compounds that can scavenge free radicals potentially ameliorate the liver injury [1,2]. Natural products and their purified compounds have received much attention as an alternative solution to numerous health problems as antioxidant agents in recent years [3]. Their antioxidant potential inhibits the generation of free radicals and exhibits significance in providing protection against hepatic damage [4].

**Figure 1:**
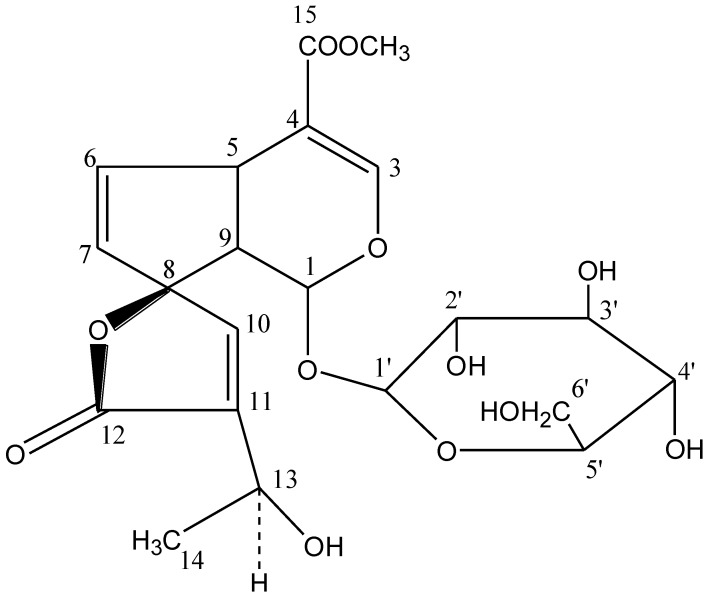
Chemical structure of plumieride.

Antioxidants and radical scavengers have been employed to study the mechanism of carbon tetrachloride (CCl_4_) toxicity as well as to protect liver cells from CCl_4_-induced damage by breaking the chain reaction of lipid peroxidation [5]. Plumieride (Figure 1) is an iridoid, isolated as a major constituent from the extract of *Plumeria bicolor* stem bark [6,7]. Further, *in vitro* anticancer potential and various pharmacological activities of plumieride have been conducted on mammals [8] whereas its antioxidant potential through possible mechanisms of action remains uninvestigated [9,10,11,12,13]. These medicinal properties and lack of references motivated us to investigate the antioxidant potential of plumieride from *Plumeria bicolor*. Hence, the present study aimed to investigate the natural antioxidant potential of plumieride, using CCl_4_-induced peroxidative damage in the liver of rats.

## 2. Materials and Methods

### 2.1. Animal Model

Adult, male, wistar albino rats (*Rattus norvegicus*) weighing 150–170 g were used in the present study. The animals were kept in standard laboratory conditions and maintained on rat diet (Lipton India Ltd., Bangalore, India) and tap water *ad libitum* under a natural light-dark cycle.

### 2.2. Plant Material

The plant material (stem bark of *Plumeria bicolor*) was collected from the campus, University of Rajasthan, Jaipur and authenticated by the Department of Botany, University of Rajasthan, Jaipur, India (Herbarium Sheet No. RUBL-20603).

### 2.3. Plant Extraction and Isolation of Active Compounds

The bark of the plant was shade dried and grinded to powder. Four kg of powdered bark was extracted with methanol (72 h). The methanol extract was filtered and evaporated to dryness under reduced pressure in a rotary evaporator (Labmate (Asia) Pvt. Ltd., Chennai, India) at <40 °C, which yielded a semi-solid brown mass. The brown mass was treated with acetonitrile for the removal of fats (acetonitrile soluble fraction). Acetonitrile was removed under reduced pressure. Fat free extract was again extracted with benzene. The benzene soluble portion afforded 40 g of crude extract after the removal of solvent. The crude extract so obtained was subjected to column chromatography. For this purpose a column of height 1.2 m with diameter 5 cm filled with 800 g Si-gel G (60–120 mesh) was taken. The column was chromatographed with various solvents. The column was eluted with different solvents using mixtures of benzene-chloroform-methanol in order of increasing polarity. The P_1_–P_8_ fractions were obtained.

### 2.4. Purification, Identification and Structure Elucidation of Active Compound

The column was eluted with chloroform and methanol in the ratio of 7:3, the purified compound-A was obtained from fraction P_8_ as colourless bright needles, 4.0 g, melting point—227 to 229 °C. The structure of purified compound-A was identified by mass spectrometry (Agilent 1100 Series LC/MSD API-ES spectrometer, Agilent Technologies Inc., Santa Clara, CA, USA), infrared analyses (FTIR-8400S Spectrophotometer, Shimadzu, Japan) and Nuclear Magnetic Resonance (NMR) (JEOL AL-300 MHz Spectrometer, JEOL USA Inc., Peabody, MA, USA.), using ^1^H NMR (300.40 MHz) and ^13^C NMR (75.45 MHz) analyses in pyridine-d_5_, respectively.

The mass spectrum of compound-A showed characteristic ion at 471 (M^+^H) and the molecular ion peak was considered to be 470 (M^+^). On the basis of ^1^H NMR spectrum, the number of protons was calculated as 26. The ^13^C NMR spectrum indicated the presence of 21 carbon atoms. On the basis of these observations, the molecular formula was assigned as C_21_H_26_O_12_.

MS m/z: 470 (M^+^), 471 (M^+^H), 461, 329, 309, 291, 273, 259, 241, 231, 213, 176, 154, 136, 107, *etc*.

In IR spectrum (KBr, cm^−1^) of the compound-A_,_ the presence of hydroxyl group was confirmed by the absorption at 3350–3200 as a broad band. The presence of α, β unsaturated carbonyl group was indicated at 1750–1742 and the presence of C=C was confirmed at 1655–1640. The absorbance at 1040 was characterized for –C-O stretching.

IR (ν_max_) cm^−1^ (KBr): 3350, 3200, 1750, 1742, 1655, 1640, 1040.

In the proton NMR spectrum (δ, ppm, pyridine-*d*_5_), a doublet observed at 1.61 (*J* = 6 Hz) was assigned for three protons of methyl group at C-14. The double doublet observed at 3.06 was assigned for one proton each 9-H. At 5.32 (*J* = 7 Hz) a doublet was observed for the 1′-H proton and a multiplet at the range of 3.80–4.50 was assigned for the proton present on the β-d-glucopyranose moiety. The two doublets at 5.40 and 6.44 were characteristic to that of protons at C-7 and C-6 of iridoid’s skeleton. On the basis of these doublets, compound-A should be a Δ^6^ iridoid. Other olefinic proton present at C-10 and C-3 were observed at 7.90 and 7.60, respectively. Absorption signal at 5.32 as a doublet were assigned for proton at C-1′ carbon atom. Thus, the nature and position of all the protons were established.

^1^H NMR (δ, ppm, pyridine-*d*_5_): 1.61 (3H, *d*, *J* = 6, 14-CH_3_), 3.06 (1H, *dd*, *J* = 6, 8 Hz, 9-H), 3.63 (3H, *s*, -COOCH_3_), 3.80–4.50 ( ~8H, m), 4.76 (1H, *dq*, *J* = 2, 6 Hz, 13-H), 5.32 (1H, *d*, *J* = 7 Hz. 1-H), 5.40 (1H, *dd*, *J* = 2, 6 Hz. 7-H), 5.58 (1H, *d*, *J* = 6 Hz, 1-H), 6.44 (1H, *dd*, *J* = 2, 6 Hz, 6-H), 7.60 (1H, *d*, *J* = 2 Hz., 3-H), 7.90 (1H, *d*, *J* = 2 Hz., 10-H).

^13^C NMR (δ, ppm, pyridine-*d*_5_): 23.00 (*q*, C-14), 40.10 (*d*, C-5), 49.90 (*d*, C-9), 51.30 (*q*, -COOCH_3_), 62.10 (t, C-6′), 62.70 (*d*, C-13), 70.70 (*d*, C-4′), 74.70 (*d*, C-2′), 78.10 (*d*, C-3′), 78.70 (*d*, C-5′), 94.00 (*d*, C-1), 96.40 (*s*, C-8), 100.80 (*d*, C-1′), 109.50 (*s*, C-4), 129.10 (*d*, C-10), 138.70 (*s*, C-11), 140.90 (*d*, C-6), 149.10 (*d*, C-7), 152.00 (*d*, C-3), 166.70 (*s*, C-15), 171.30 (*s*, C-12).

The ^13^C NMR spectrum (δ, ppm, pyridine-*d*_5_) showing a sharp signal at 166.7 was assigned for C-15. The signals at 23.0, 62.70 and 171.3 were assigned for C-14, C-13 and C-12 carbon atoms, respectively. The C-1 carbon atom to which glucose moiety was attached shows absorbance at 94.0. The presence of six olefinic carbon atoms was confirmed by the absorptions at 152.00 (C-3), 109.50 (C-4), 40.1 (C-5), 140.9 (C-6), 149.10 (C-7), 96.40 (C-8), 49.9 (C-9), 129.1 (C-10) and 138.70 (C-11).

The presence of glucose moiety was confirmed by the characteristic absorption at 100.80 for anomeric carbon (C-1′), 74.70 (C-2′), 78.10 (C-3′), 78.70 (C-4′), 78.70 (C-5′), and 62.10 (C-6′). The remainders of the absorptions were also in good agreement with the reported values for Plumieride [14,15,16]. Therefore, on the basis of ^1^HNMR, ^13^CNMR and mass spectral studies of compound-A from fraction P_8_ was characterized as plumieride (Figure 1) with molecular formula C_21_H_26_O_12_ and used for the exploration of its antioxidant potential against peroxidative damage in the liver of rats.

### 2.5. Standard Drug

Silymarin was purchased from MP Biomedicals, Strasbourg, France and it was dissolved in olive oil for oral administration to rats during experimentation at the dose level of 20 mg/kg body weight/day [1].

### 2.6. Chemicals

All chemicals were analytical grade, and chemicals required for all biochemical assays were obtained from Sigma Chemicals Co., St. Louis, MO, USA.

### 2.7. Ethical Aspects

The study was approved by the ethical committee (Protocol No: 1678/Go/a/12/CPCSEA/82) of the University Department of Zoology, Jaipur, India. Indian National Science Academy, New Delhi (INSA, 2000) guidelines were followed for maintenance and use of the experimental animals.

### 2.8. Behavioral and Toxic Effects

The pure compound- Plumieride from *Plumeria bicolor* was administered to all the test groups in graded doses ranging up to 80 mg/kg body weight/day and the rats were observed for any symptoms of mortality and behavioral toxicity for 30 days afterward. The compound was found to be practically non-toxic when given orally to rats and its LD_50_ value was found to be higher than 80 mg/kg body weight [1]. The minimum dose levels *viz.* 5, 10 and 20 mg/kg body weight were used for the oral administration to rats during experimentation [17].

### 2.9. Experimental Protocol

After acclimatization of 15 days, the animals were divided into the following groups containing six animals in each group:

Group I: Vehicle treated rats were kept on normal diet and served as control for 30 days.
Group II: Rats were intoxicated with carbon tetrachloride at 0.3 mL/kg body weight/twice a week with olive oil (1:1), intra-peritoneally for 30 days.
Group III: Rats orally received plumieride at 5 mg/kg body weight/day with olive oil, and CCl_4_ as Group II for 30 days, simultaneously.
Group IV: Rats orally received plumieride at 10 mg/kg body weight/day with olive oil, and CCl_4_ as Group II for 30 days, simultaneously.
Group V: Rats orally received plumieride at 20 mg/kg body weight/day with olive oil, and CCl_4_ as Group II for 30 days, simultaneously.
Group VI: Rats orally received silymarin at 20 mg/kg body weight/day with olive oil, and CCl_4_ as Group II for 30 days, simultaneously.

### 2.10. Assessment of Liver Function

After 24 h of last dose-delivery, the rats were anaesthetized under mild ether anesthesia. Blood samples were collected by cardiac puncture and allowed to clot at 37 °C. Serum was separated by centrifugation then stored at 4 °C until assayed. The serum-AST, ALT (aspartate and alanine transaminases), GGT (gamma glutamyl transpeptidase), ALP (alkaline phosphatase), total bilirubin and total protein were determined using diagnostic kits. AST (batch No. 61105), ALT (batch No. 60805), GGT (batch No. 34004) kits were purchased from Accurex Biomedical Pvt. Ltd., Mumbai, India, as well as ALP (lot No. 7093), total bilirubin (lot No. 6801), and total protein (lot No. 6808) kits were purchased from Span Diagnostic Ltd., Surat, India.

After the collection of blood, the liver was immediately excised, washed with cold normal saline, blotted and weighed on an electrical balance. Half of the liver was fixed in Bouin’s fixative for histological studies and remaining half was immediately frozen (at −20 °C/−70 °C) for biochemical analysis. After that, a part of it was minced and homogenized in ice-cold 1.15% ^w^/_v_ KCl in a Potter Elvehjem Teflon glass homogenizer for 1 min to make a 10% ^w^/_v_ liver homogenate. The lipid peroxidation (LPO) [18] was measured in the liver homogenate. The activities of hepatic antioxidant enzymes such as SOD (superoxide dismutase) [19],CAT (catalase) [20], GSH (reduced glutathione) [21], GR (glutathione reductase) [22] and GPx (glutathione peroxidase) [23] were also determined, respectively.

### 2.11. Histopathology

Liver was fixed in Bouin’s fixative for 24 h then dehydrated in ethanol series (50%–100%), cleared in xylene, and embedded in paraffin using the standard microtechnique. Sections of the liver (5 μm) were stained with alum haematoxylin and eosin (H & E) for histopathological changes.

### 2.12. Statistical Process

Statistical analysis was performed using one-way analysis of variance (ANOVA) followed by student *t*-test. The values were mean ± S.E. for six rats in each group. *p*-value ≤ 0.05 were considered as significant.

## 3. Results

The results of antioxidant potential of plumieride in CCl_4_-intoxicated rats are shown in Table 1 and Table 2. CCl_4_-intoxication to rats for the period of 30 days resulted in a significant (*p* ≤ 0.001) increase in the levels of AST, ALT, GGT, ALP and total bilirubin along with a remarkable reduction (*p* ≤ 0.001) in the level of total proteins in serum when compared with normal controls. The effect of plumieride on the serum- AST, ALT, GGT, ALP and total bilirubin in CCl_4_-induced rats was found to be dose-dependently significant (*p* ≤ 0.05; *p* ≤ 0.001) reduction along with the remarkable elevation in the total proteins at all three dose levels (Groups III–V) as compared to CCl_4_-treated Group-II. The degree of protection by plumieride (20 mg/kg) was observed statistically similar to the silymarin treated Group VI (Table 1).

Rats treated with CCl_4_, caused a significant (*p* ≤ 0.001) decline in the hepatic antioxidants such as SOD, CAT, GSH, GPx and GR in comparison to normal controls. Simultaneously, oral treatment with plumieride at all three dose levels (Groups III–V) showed significant (*p* ≤ 0.05; *p* ≤ 0.001) elevation in the activity of all antioxidant parameters such as SOD, CAT, GSH, GPx and GR. The elimination of hepatic oxidative stress by plumieride (Group V) and silymarin (Group VI) was statistically almost similar in nature (Table 2).

**Table 1:** Showing antioxidant potential of plumieride and silymarin against CCl_4_-induced peroxidative damage in rat liver through serum parameters.

Treatment Design	AST (IU/L)	ALT (IU/L)	GGT (IU/L)	ALP (KAU)	Total Bilirubin (mg/100 mL)	Total Protein (mg/dL)
Control (vehicle treated) Group I	122.27 ± 1.37	104.33 ± 2.45	8.24 ± 0.28	19.37 ± 1.19	0.82 ± 0.05	6.32 ± 0.29
CCl_4_ (0.3 mL/kg body weight/twice a week with olive oil, intra-peritoneally, 1:1) Group II	356.45 ± 3.42 ***	330.72 ± 4.57 ***	43.92 ± 2.10 ***	65.33 ± 2.19 ***	2.85 ± 0.14 ***	2.69 ± 0.14 ***
CCl_4_ + plumieride (5 mg/kg body weight/day, orally) Group III	275.50 ± 2.66 ^a^	262.14 ± 2.59 ^a^	35.10 ± 1.35 ^b^	56.48 ± 1.59 ^b^	2.10 ± 0.15 ^b^	3.12 ± 0.15 ^ns^
CCl_4_ + plumieride (10 mg/kg body weight/day, orally) Group IV	224.26 ± 1.98 ^a,c^	201.21 ± 1.92 ^a,c^	28.05 ± 0.72 ^a,c^	48.10 ± 1.37 ^a,d^	1.75 ± 0.16 ^a,ns^	4.56 ± 0.10 ^a,c^
CCl_4_ + plumieride (20 mg/kg body weight/day, orally) Group V	172.24 ± 1.49 ^a,c,e^	157.20 ± 1.37 ^a,c,e^	19.10 ± 0.65 ^a,c,e^	35.15 ± 1.18 ^a,c,e^	1.27 ± 0.12 ^a,d,f^	6.42 ± 0.17 ^a,c,e^
CCl_4_ + silymarin (20 mg/kg body weight/day, orally) Group VI	145.18 ± 1.55 ^a,c,e,g^	128.37 ± 1.92 ^a,c,e,g^	15.75 ± 1.77 ^a,c,e,h^	30.47 ± 1.72 ^a,c,e,i^	1.22 ± 0.08 ^a,c,f,ns^	6.19 ± 0.18 ^a,c,e,ns^

Levels of significance; Data are mean ± SEM (*n* = 6); *** *p* ≤ 0.001, Group II compared with control (Group I); ^a^ *p* ≤ 0.001; ^b^ *p* ≤ 0.01; ^ns^ non-significant, Groups III–VI compared with Group II; ^c^ *p* ≤ 0.001; ^d^ *p* ≤ 0.01; ^ns^ non-significant, Groups IV–VI compared with Group III; ^e^ *p* ≤ 0.001; ^f^ *p* ≤ 0.05, Groups V & VI compared with Group IV; ^g^ *p* ≤ 0.001; ^h^ *p* ≤ 0.05; ^i^ *p* ≤ 0.01; ^ns^ non-significant, Group VI compared with Group V.

**Table 2:** Showing antioxidant potential of plumieride and silymarin against CCl_4_-induced peroxidative damage in rat liver through tissue parameters.

Treatment Design	LPO (*n* mol TBARS/mg Tissue)	SOD (μmol/mg Protein)	CAT (μmol H_2_O_2_ Consumed/min/mg Protein)	GSH (*n* mol/g Tissue)	GPx (*n* mol NADPH Consumed/min/mg Protein)	GR (*n* mol NADPH Consumed/min/mg Protein)
Control (vehicle treated) Group I	3.04 ± 0.21	10.24 ± 0.42	62.22 ± 2.33	4.52 ± 0.19	12.18 ± 0.42	18.27 ± 0.62
CCl_4_ (0.3 mL/kg b. wt/twice a week with olive oil, intra-peritoneally, 1:1) Group II	11.23 ± 0.89 ***	4.10 ± 0.13 ***	26.21 ± 1.94 ***	1.72 ± 0.09 ***	5.09 ± 0.25 ***	8.82 ± 0.15 ***
CCl_4_ + plumieride (5 mg/kg body weight/day, orally) Group III	8.18 ± 0.32 ^a^	5.32 ± 0.14 ^a^	32.10 ± 1.52 ^b^	2.05 ± 0.10 ^b^	6.62 ± 0.21 ^a^	10.52 ± 0.17 ^a^
CCl_4_ + plumieride (10 mg/kg body weight/day, orally) Group IV	6.22 ± 0.29 ^a,c^	6.89 ± 0.16 ^a,c^	40.21 ± 1.89 ^a,d^	3.06 ± 0.14 ^a,c^	ai8.35 ± 0.18 ^a,c^	12.82 ± 0.22 ^a,c^
CCl_4_ + plumieride (20 mg/kg body weight/day, orally) Group V	3.95 ± 0.35 ^a,c,e^	7.83 ± 0.18 ^a,c,f^	48.58 ± 1.23 ^a,c,f^	3.95 ± 0.13 ^a,c,e^	10.10 ± 0.15 ^a,c,e^	16.17 ± 0.21 ^a,c,e^
CCl_4_ + silymarin (20 mg/kg body weight/day, orally) Group VI	3.68 ± 0.42 ^a,c,e,ns^	8.14 ± 0.33 ^a,c,f,ns^	51.40 ± 1.67 ^a,c,f,ns^	3.63 ± 0.12 ^a,c,e,ns^	10.21 ± 0.24 ^a,c,e,ns^	15.28 ± 0.33 ^a,c,e,g^

Levels of significance, Data are mean ± SEM (*n* = 6); *** *p* ≤ 0.001, Group II compared with control (Group I); ^a^ *p* ≤ 0.001; ^b^ *p* ≤ 0.05, Groups III–VI compared with Group II; ^c^ *p* ≤ 0.001; ^d^ *p* ≤ 0.01, Groups IV–VI compared with Group III; ^e^ *p* ≤ 0.001; ^f^ *p* ≤ 0.01, Groups V & VI compared with Group IV; ^g^ *p* ≤ 0.05; ^ns^ non-significant, Group VI compared with Group V.

Table 2 illustrates a significant (*p* ≤ 0.001) increase in the level of hepatic LPO in CCl_4_-intoxicated rats as compared to normal control. In contrast, treatment with plumieride at all three dose levels (Groups III–V) showed a significant (*p* ≤ 0.05; *p* ≤ 0.001) lowering effect on CCl_4_-induced elevation of hepatic LPO contents. The lowering effect of LPO by plumieride (Group V) and silymarin (Group VI) was found to be statistically equal against CCl_4_-induced lipid peroxidation (Table 2).

**Figure 2:**
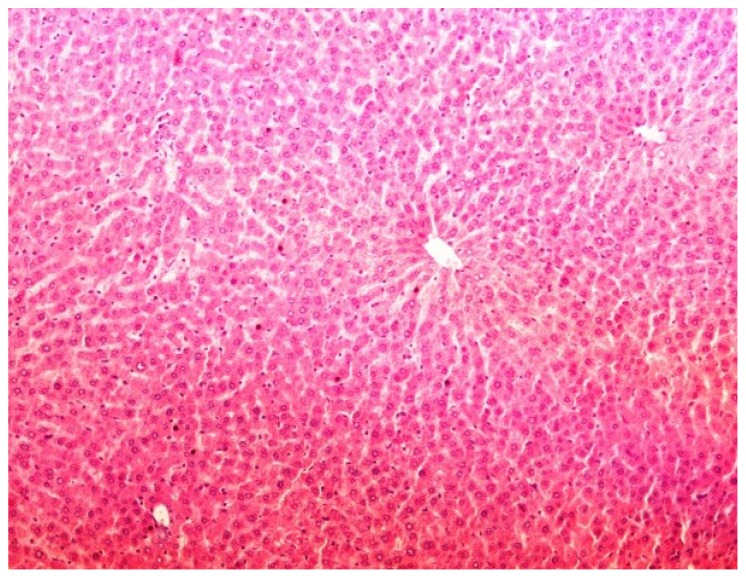
Photomicrograph of control rat liver section showing well brought central vein, hepatic cells with preserved cytoplasm and prominent nucleus at H & E × 10.

**Figure 3:**
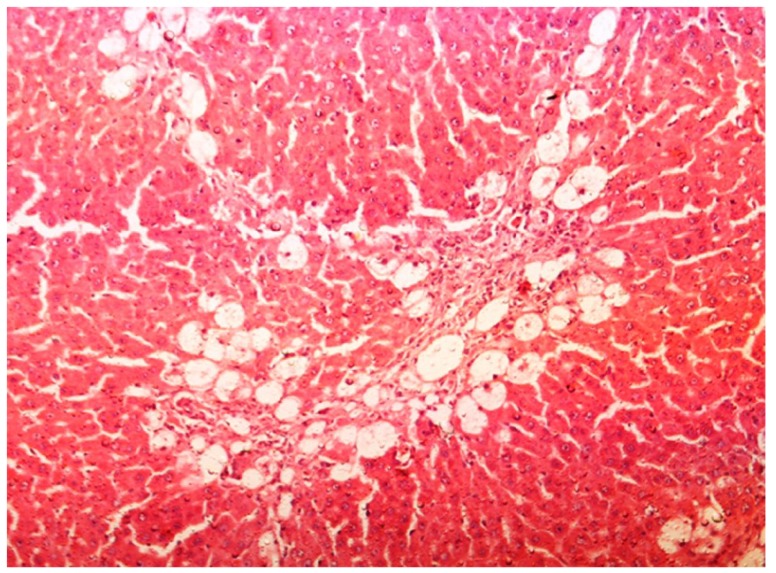
Photomicrograph of rat liver section with CCl_4_ treatment showing ballooning degeneration and distended portal vein, mild periportal fibrosis and necrosis at H & E × 100.

**Figure 4:**
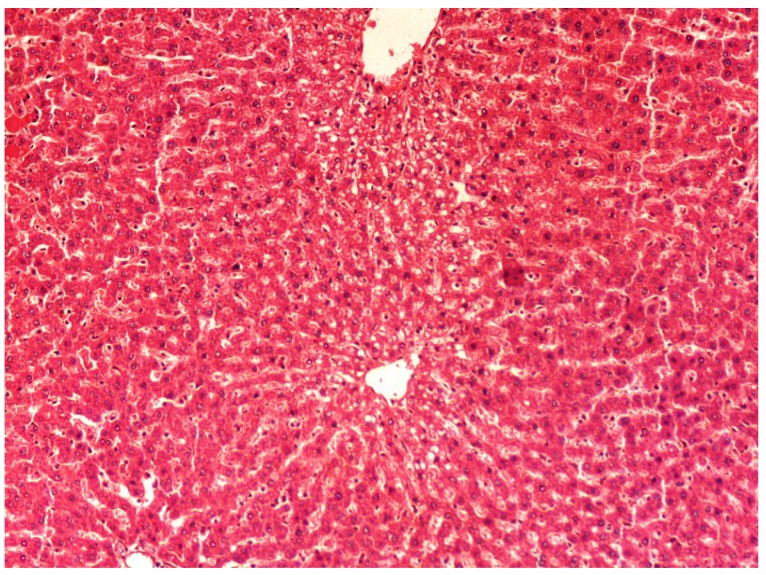
Photomicrograph of rat liver section of CCl_4_ + Plumieride (5 mg/kg body weight), showing reasonable reduction in necrosis, fatty changes along with cytoplasmic clearing at H & E × 100.

**Figure 5:**
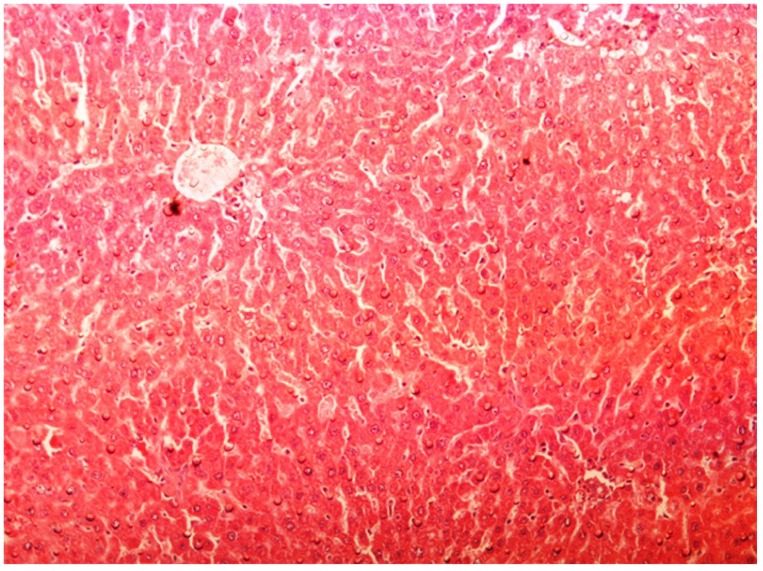
Photomicrograph of rat liver section of CCl_4_ + Plumieride (10 mg/kg body weight), reflecting considerable reduction in necrosis, fatty changes and exhibiting cytoplasmic clearing at H & E × 100.

**Figure 6:**
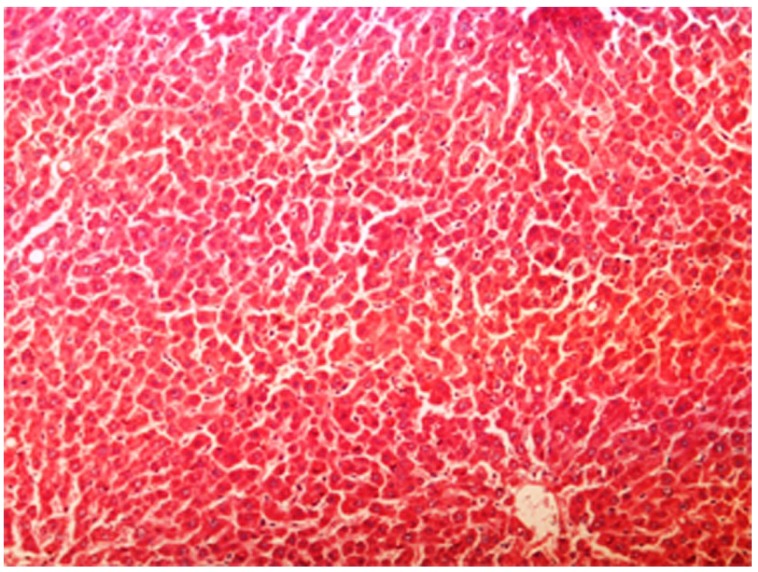
Photomicrograph of rat liver section of CCl_4_ + Plumieride (20 mg/kg body weight), showing moderately brought central vein, hepatic cells with preserved cytoplasm and prominent nucleus at H & E × 100.

**Figure 7:**
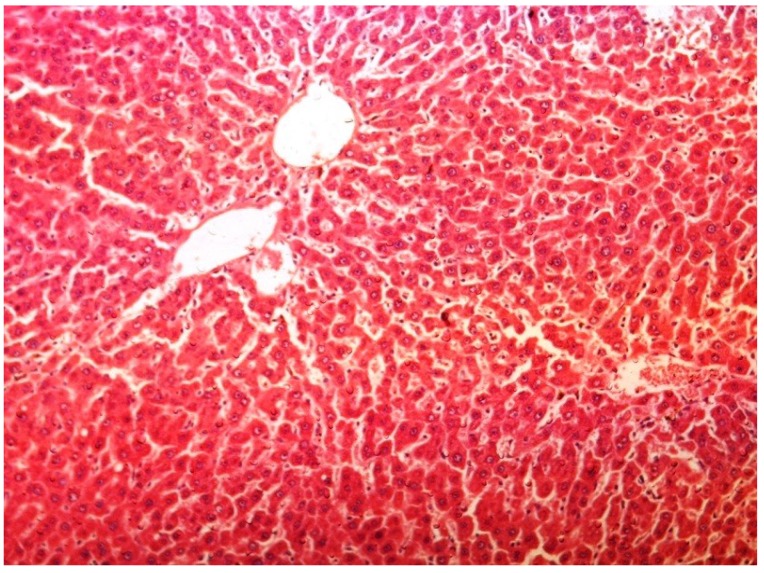
Photomicrograph of rat liver section of CCl_4_ + Silymarin (20 mg/kg body weight), showing moderately brought central vein, hepatic cells along with preserved cytoplasm and prominent nucleus at H & E × 100.

The histopathology of CCl_4_-induced rats when compared to normal hepatic architecture (Figure 2) showed massive fatty changes, necrosis, ballooning degeneration and the loss of cellular boundaries (Figure 3). The liver sections of plumieride at the dose level of 5 mg/kg body weight plus CCl_4_-treated rats (Group III) showed mild prevention of CCl_4_-induced degenerative changes with the few pyknotic nuclei and fatty vacuolizations in the cytoplasm (Figure 4). The liver sections of plumieride treated rats at the dose level of 10 mg/kg body weight along with CCl_4_ (Group IV) indicated partial amelioration of degenerative effects in hepatocytes but still show cloudy swelling and mild fatty changes (Figure 5). The histomorphological picture of liver sections of plumieride at the dose level of 20 mg/kg body weight along with CCl_4_-induction to rats (Group V) showed more or less normal labular patterns devoid of degenerative changes, and cytoplasm was preserved with prominent nucleus without intracellular lipid accumulation (Figure 6) almost comparable to the normal control and silymarin treated Group VI (Figure 7).

## 4. Discussion

Reactive oxygen species (ROS) are known for their role in a large number of diseases including hepatic injury because hepatic cells appear to participate in a variety of enzymatic metabolic activities. The natural antioxidant mechanisms of the body can be insufficient and dietary intake of additional antioxidant constituents appear to be necessary [24,25].

CCl_4_-induced hypofunctions of the hepatic cell membrane due to hepatic injury are associated with the peroxidation of lipids and reduced antioxidant levels through the production of toxic species—trichloromethyl free radical (CCl_3_^•^) or/and trichloromethyl peroxy radical (CCl_3_OO^•^) which in turn alter the hepatic metabolism via oxidative stress leading to hepatotoxicity [26,27].

The lipid peroxidative degradation of biomembranes is one of the principle causes of hepatotoxicity induced by CCl_4_ [4,28]. This is evidenced by the elevation of serum marker enzymes such as AST, ALT, GGT, ALP along with the total bilirubin and reduction in the level of total protein. This is indicative of cellular leakage and loss of functional integrity of cell membrane of the liver tissue [29], concomitantly with the alterations in biliary flow which reflects ALP with the high level of total serum bilirubin contents [30], and finally inhibits the protein synthesis due to trichloromethyl free radical covalent bindings [31]. Reduction in the levels of AST, ALT, GGT and ALP towards the respective normal values by the oral treatment of plumieride and silymarin is an indication of the stabilization of plasma membranes as well as repair of hepatic tissue damage caused by CCl_4_ [1,32]. Further, the depletion of raised total bilirubin concentration suggests the possibility of the plumieride being able to stabilize the biliary dysfunctions and improvement of functional status of the liver cells [33]. Additionally, the stabilization of total proteins might be considered as an indicator of the improvement of total protein synthesis in the hepatic cells due to the inhibition of peroxidation of lipids and scavenge the free radicals [34]. This indicates the anti-lipid peroxidation and/or adaptive nature of the systems brought about by plumieride against the damaging effects of free radicals produced by CCl_4_.

The LPO is accelerated when free radicals are formed as the result of losing a hydrogen atom from the double bond in the structure of unsaturated fatty acids. Scavenging of free radicals is one of the major antioxidant mechanisms to inhibit the chain reaction of LPO [1,4].

Plumieride restores the antioxidant status by its ability to inhibit the peroxidation of membrane lipids and maintains cell membrane integrity and function. The presence of hydrogen ions in the alcoholic group(s) of plumieride might be responsible for the inhibitory effect, and also the oxygen belonging to the furanic ring might be able to develop a resonance property which is merely a change in the redistribution of electrons, effectively neutralizing them.

SOD and CAT are easily inactivated by lipid peroxides or reactive oxygen species which results in decreased activities of these enzymes in CCl_4_ hepatotoxicity [35,36]. The hepatic GSH, GPx and GR play the primary role in protection against trichloromethyl radical-induced liver damage. Kojima *et al.* [37] have also reported that under oxidative stress, GSH is largely consumed by the glutathione-related enzymes thereby resulting in intoxication of toxic metabolites. Therefore, it has been suggested that lipid peroxides generated after CCl_4_ exposure is eliminated by GPx and GR in the presence of GSH, resulting in the decreased propagation of lipid peroxidation [5,38]. Administration of plumieride and silymarin showed antioxidant potential by scavenging the endogenous metabolic peroxides generated after CCl_4_ induction to rats, which resulted in an increase in the activities of tissue enzymatic antioxidants and glutathione concentration.

Histopathological observations suggested that the ROS and LPO may play an important role in the pathogenesis of hepatocytes as hepatic fibrosis and necrosis with the loss of normal liver architecture. Because of CCl_4_ toxicity, a toxic reactive metabolite-trichloromethyl free radical was produced which binds covalently to the macromolecules of the lipid membranes of the adipose tissue and causes peroxidative degradation. As a result, fats from the adipose tissue are translocated and accumulated in the hepatocytes [1,5,39].The degenerative changes showed minimal or absence with plumieride and silymarin treatments. Plumieride, because of low fat accumulation, can scavenge free radicals, interact with oxidative cascade, quench oxygen, chelate metal ions, inhibit oxidative enzymes and LPO; finally, it can restore the antioxidant status of hepatic tissues.

## 5. Conclusions

Based on our observations, plumieride consists of sugar molecules, which have alcoholic groups. The alcoholic groups of sugar seems to increase its antioxidant potential through intermolecular hydrogen bonding along with the thiol (SH) group of non-protein thiols and enzymes resulting in the restoration of the antioxidant system against peroxidative damage. It may also stimulate hepatic regeneration through an improved synthesis of proteins, or due to its ability to block the bioactivation of CCl_4_, by inhibiting the P450 2E1 activity and/or its accelerated detoxification, as well as having the potential to minimize the deleterious effects of free radicals including the peroxy radicals and its antioxidant activity in combination with inhibition of LPO. In summary, plumieride might be considered a natural antioxidant agent against peroxidative damage in mammalian liver tissue.
